# New Memoirs of the Academy of Dijon, Relative to the Sciences and Arts

**Published:** 1784

**Authors:** 


III. *Nouveaux Memoires de l'Academie de Dijon, pour la partie des Sciences et Arts*; i. e. *New Memoirs of the Academy of Dijon, relative to the Sciences and Arts.* Vol. I. for 1782. Part II. 8vo. Dijon, 1783.

1. *ON the means of saturating the mother waters of nitre without loss of alkali; and of avoiding the mixture of the salt of Sylvius with the saltpetre. By* M. de Morveau.—2. *Sequel to the Essay*

*Eſſay on biliary concretions. By* M. Durande. (ſee our 4th vol. p. 381).—Eight caſes are here related, in which a mixture of æther and ſpirit of turpentine was adminiſtered with ſucceſs. The reader will find an account of this remedy in a former part (p. 126) of our preſent volume.—3. *Account of the lead mine found at Saint Prix ſous Beuvray, with mineralogical obſervations on that part of Burgundy. By* Meſſieurs *de* Morveau *and* Champy.—4. *Aſtronomical obſervations, by* M. Roger.—5. *On the glow-worm. By* M. Guenau de Montbeillard.—6. *Analyſis of the mineral waters of Premeaux in Burgundy. By* M. Maret.—7. *Obſervations relative to natural hiſtory, made in croſſing the province of Burgundy, from the Yonne to the Saone, that is, from Auxerre to Chalons; to which are added ſome philoſophical remarks. By* M. Pazumot.—8. *Caſe of a colic occaſioned by biliary concretions, and cured by the ſolvent of thoſe Calculi. By* M. Maret.—The patient, whoſe caſe is here related, was a man ſixty years old, of a robuſt habit, and ſubject to frequent attacks of colic, accompanied with jaundice. Theſe ſymptoms were aſcribed to biliary concretions, and recourſe was had to M. Durande's method of treatment. At firſt the patient took the Solvent once, and afterwards wards twice a day, in the doſes recommended by M. Durande, and during the uſe of this remedy he drank plentifully of diluents, and avoided all ſtrong liquors. Before he had taken two ounces of the medicine his jaundice had in a great meaſure diſappeared, and his urine and fœces had almoſt entirely reſumed their natural colour. By the time he had taken another ounce of it, he found himſelf perfectly cured, and when this account was written he had remained well ſix months. No gall-ſtones were obſerved in his ſtools. M. Maret adds, that he could mention other inſtances of the efficacy of this Solvent.—9. *Farther remarks on ſluices. By* M. Gauthey.—10. *Extract from the meteorological diary kept by M. Maret from the year* 1763 *to the preſent.*—11. *Natural and botanic hiſtory of Cevadilla. By* M. Willemet. - Monard, a phyſician of the 16th century, was the firſt who mentioned this plant, of which he has given a wooden cut in his *Hiſtoria ſimplicium Medicamentorum ex novo Orbe delatorum.* He ſpeaks of its cauſtic qualities; but obſerves that its ſtrength may be moderated by infuſing it in roſe-water. Lemery, who has copied from this writer what he ſays of the Cevadilla in his

Dictionary

Dictionary of Drugs, obſerves that, in his time, none of it was to be ſeen in France, but at preſent, we are told, its ſeeds and capſula are very common in trade. The plant that produces them is not yet ſatisfactorily aſcertained. Linnæus, tho' he ſpeaks of its acrid and poiſonous properties in his Materia Medica, has not been able to claſs it in his ſexual ſyſtem. Haller claſſes it with the *Delphinium conſolida* and *Aconitum*. An herboriſt of Lorraine has aſſured our author, that it is no other than the yellow aconite, and he is endeavouring, he tells us, to aſcertain the truth of this by attempting to make the ſeeds of Cevadilla vegetate. The moſt ſatisfactory account, which Mr. Willemet has met with of the natural hiſtory of the plant in queſtion, is in the botanic obſervations of Retzius, publiſhed at Leipſic in 1779. The powder of Cevadilla forms the baſis of a compoſition called *Capuchins Powder* (*Poudre des Capuchins*) uſed in France for deſtroying lice, and this, we are told, is the only uſe to which it is at preſent applied. —12. *Remainder of the meteorological hiſtory of* 1782. *By* Maret.

IV. *Be-*

